# READMISSION FOR HEALTHCARE-ACQUIRED INFECTIONS: DOES PATIENT DISPOSITION MATTER?

**DOI:** 10.1093/geroni/igz038.3030

**Published:** 2019-11-08

**Authors:** Geoffrey J Hoffman, Lillian Min, Haiyin J Liu, Lona Mody

**Affiliations:** University of Michigan School of Nursing, Ann Arbor, Michigan, United States

## Abstract

Both common and preventable, healthcare-acquired infections (HAI) are nevertheless associated with high risk for hospital readmission. However, whether these infection-related readmissions are more common among older adults discharged from the hospital to a nursing facility as opposed to home is unknown. We used 2013-14 HCUP data and multivariable logistic regression models to retrospectively examine the relationship of patient disposition (home, nursing facility, home health care) with an unplanned readmission for the same HAI observed at the index admission, among older Medicare beneficiaries, controlling for patient sociodemographics, comorbidity score, and length of stay during index hospitalization. Of 8.4 million index admissions, 323,332 (3.9%) involved an index HAI, of which 15,870 (4.9%) resulted in a linked HAI readmission. HAI readmissions were more common for Clostridium difficile infections (4.0%) and urinary tract infections (UTI, 2.3%) than for ventilator-acquired pneumonia (1.4%) or surgical site infections (1.1%) (p<0.001). Being discharged home or to home health care, compared to a post-acute care setting, was associated with increased odds (OR: 1.63 and 1.62, p<0.001) of HAI readmission, particularly for patients with higher comorbidity scores. For home discharges, HAI readmission risk was doubled for patients with the most compared to fewest comorbidities while nursing facility discharges were equally protective across comorbidity levels. We conclude that Clostridium difficile and UTIs result in higher risk for readmission than other HAIs. Patients discharged to nursing facilities are protected from readmission. Further research into identifying modifiable mechanisms for HAI readmission, in order to improve post-hospital care of infection at home, is needed.

